# Impact of Healthcare Access Disparities on Initial Diagnosis of Breast Cancer in the Emergency Department

**DOI:** 10.7759/cureus.10027

**Published:** 2020-08-25

**Authors:** Allison M Yee, Preeanka K Mazumder, Fanglong Dong, Michael M Neeki

**Affiliations:** 1 Emergency Medicine, Arrowhead Regional Medical Center, Colton, USA; 2 Emergency Medicine, California University of Science and Medicine, San Bernardino, USA

**Keywords:** breast cancer, healthcare disparities, emergency department, hispanic, african american, initial diagnosis, diagnostic delay

## Abstract

Breast cancer continues to be the second leading cause of cancer deaths in women in the United States. This is more noticeable in communities with pronounced healthcare disparities. The aim of this study was to investigate the different demographics that might play a role in the detection of breast cancer in a county hospital emergency department (ED).

A retrospective study was conducted of female patients diagnosed with breast cancer over a five-year period (1/1/2015 to 12/31/2018). Patients with breast cancer as the primary or secondary diagnosis were identified.

This study shows that 66 (73.3%) women diagnosed in the ED were Hispanic or African American. There was a significant delay (a median of 461 days) in the time between the diagnosis of suspected breast cancer in the ED to their follow-up visit with definitive diagnosis in a primary care clinic.

These findings suggest that women with a suspected breast cancer diagnosis who are seen in a safety net hospital and have Medicaid funding may have significant delays before final diagnosis is made. Patient demographics could have an impact on the patients' access to screening and regular healthcare visits, hindering an early breast cancer diagnosis by a primary care provider.

## Introduction

Breast cancer is the most common type of cancer in women worldwide. In the United States (U.S.), there were 245,299 newly diagnosed breast cancer cases and 41,487 deaths in 2016 [1]. Because of the health care burden due to breast cancer, screening is recommended for asymptomatic adults. The U.S. Preventive Services Task Force (USPSTF) recommends that all women ages 50 to 74 years old receive screening mammography every two years [1,2]. However, women who are below the age of 50 years or who do not have access to breast cancer screening mammography are a fallout risk. In addition, without access to a primary care provider and routine early screening, these women of low socioeconomic status or marginalized ethnicity may present with various stages of breast cancer at the time of diagnosis in the emergency department [3].

Several demographic factors are known to influence the stage of cancer at diagnosis and are linked to a worse prognosis. Patients diagnosed with late-stage breast cancer were more likely to be nursing home residents or those who were not enrolled in Medicaid prior to their diagnosis [4]. Ramirez et al. found that advanced age was a statistically significant predictor of patient delay [5]. Several studies have shown that low socioeconomic status, lack of insurance, race, cultural differences, and marital status were also significant factors for a diagnostic delay [6-8]. Although multiple studies have examined the demographics of patients diagnosed with late-stage breast cancer in a primary care setting, none have examined the demographics of women newly diagnosed with breast cancer in the emergency department (ED).

This study aims to identify common demographics and possible health care disparities of women initially diagnosed with breast cancer in a large urban safety net hospital ED located in San Bernardino County. This hospital has seen a higher number of patients presenting to the ED with a new diagnosis of advanced breast cancer, indicating a poor prognosis. Awareness of common threads within this population of patients could help shape the formation of better diagnostic algorithms for patients presenting to the ED with a first-time diagnosis of breast cancer.

## Materials and methods

We conducted a retrospective study in the ED that included patients seen at Arrowhead Regional Medical Center (ARMC) from 1/1/2015 to 12/31/2018. The institutional review board at ARMC approved this study. ARMC is a 456 bed-acute care teaching facility owned and operated by San Bernardino County in California [9]. San Bernardino County has an estimated population size of 2,180,085 and is the largest common threads county in the U.S. with an area of 20,105 square miles. The largest race of San Bernardino County residents is Hispanic at 51%. The other races of residents are White (31%), Asian (8%), Pacific Islander (8%), Black/African American (7%), two or more races (2%), and Native American (0.4%). The median household income is $63,857 and 11.7% of families are living in poverty, which is higher than the state and national averages. The percentage of uninsured residents in San Bernardino County is 8.7%, and 30.8% of residents are covered under Medicaid [10]. Of the patients presenting to the ED at ARMC during that period, 9% were uninsured and 65% had government-funded insurance. The racial demographics of the patients who visited the ED were 64% Hispanic, 17% White, 15% Black/African American, 2% Asian, and 2% Unknown, Native American, or Other.

Patients included in this study were female, ages 18 and older, and treated in the ED from 1/1/2015 to 12/31/2018. Patients diagnosed with breast cancer as the primary or secondary diagnosis were identified using International Classification of Disease, Ninth Revision (ICD-9) CODE 174 (174, 174.8, 174.9, C50.119, C50.412, C50.911, C50.912, C50.919, D05.11, D05.12, D05.91, D05.92, N61, Z85.3). Patient demographics in this study were abstracted from the medical record and included race, marital status, and insurance status. Marital status was classified as married or single, which was defined as unmarried, divorced, separated, or widowed. The median time from ED initial diagnosis of breast cancer to family health clinic follow-up was determined based on a review of electronic health records.

All statistical analyses were conducted using the SAS software for Windows version 9.3 (Cary, NC, USA). Descriptive statistics were presented as means and standard deviations for continuous variables, along with frequencies and proportions for categorical variables.

## Results

A total of 90 patients suspected to have a diagnosis of breast cancer in the ED and led to a confirmed diagnosis of breast cancer by the family medicine physician were identified between 1/1/2015 to 12/31/2018. The average age at time of diagnosis was 54.9 (SD=10.5) years. All 90 patients were eventually covered by California's version of Medicaid, a government-subsidized health insurance for low-income families or individuals. The median time from ED initial diagnosis of breast cancer to family health clinic follow-up was 461 days (first quartile was 138, third quartile was 732).

**Table 1:** Number of patients diagnosed in the emergency department (ED) with breast cancer sorted by year, race, marital status, and insurance type.

ED visit year	Frequency	Percent
2015	13	14.4
2016	34	37.8
2017	27	30.0
2018	16	17.8
Race		
White	16	17.8
African American	20	22.2
Hispanic	46	51.1
Other (combination of Asian, Pacific Islander, and unknown race)	8	8.9
Marital Status		
Married	28	31.1
Single, divorced, or widowed	62	68.9
Insurance		
Medicaid	90	100.0

## Discussion

Breast cancer is the second leading cause of cancer deaths among all women worldwide and the leading cause among Hispanic women [11]. At ARMC, most of the patient population is composed of Hispanic individuals who receive Medicaid and other publicly funded health plans. This study’s primary focus was to explore the demographics, including marital status, insurance status, and race, of breast cancer patients during evaluation in the ED at ARMC and have a confirmation of breast cancer by the primary care provider. Insight into the demographics that might be associated with a delay in detection of breast cancer could improve chances of earlier diagnosis within the San Bernardino County’s population. 

Several studies have shown that a delay in diagnosis of breast cancer greater than three months can lead to worse survival, increasing tumor size, and extensive metastatic disease [5,12,13]. Because we found a marked delay to time of definitive breast cancer diagnosis, our findings suggest that the patients diagnosed in the ED at ARMC may experience a lower survival rate than patients who are diagnosed earlier by their PCP. This delay in diagnosis may be correlated with a significant number of our patient population living in poverty, having inadequate insurance, and poor access to the recommended screening as suggested by the U.S. Preventive Services Task Force [2,10]. These barriers to healthcare prevent earlier diagnosis and treatment, and as a result, patients presenting at the ED at ARMC are more likely to have a substandard prognosis and survival rate [3,14]. In addition, Rogers et al. found that half of their patient population was diagnosed with cancer in the ED and suggested that their patients had limited awareness and knowledge about accessing PCPs even though the patients had insurance [3]. Multiple studies suggested that types of insurance matter to timely breast cancer diagnosis. Having private insurance placed patients at an advantage of easier access to quality health care as compared to patients with public health insurance [14-17]. In addition, lack of insurance or public insurance together with the patients’ poor socioeconomic status and finances create significant barriers to seeking help [6]. A similar pattern of delay from the initial diagnosis of breast cancer in the ED to follow up with primary care clinics was noted in current research. This could be due to the limited access or extended process patients must face to qualify for Medicaid. 

Current data suggest that more single women received an initial diagnosis with breast cancer in the ED than married women. This finding agrees with the literature that single women had a delay to diagnosis and worse prognosis more often than married women [8,18-20]. This observation can be attributed to the fact that single women are more likely to lack social and financial support regarding medical issues. Single women may postpone their visit to a PCP or the ED due to work, lack of transportation, or even lack of emotional support to focus on their health [20].

The Centers for Disease Control and Prevention report that Caucasians have the highest incidence of breast cancer in the U.S. A previous study conducted at ARMC suggested that Hispanics had the highest incidence of breast cancer in San Bernardino County, correlating with the racial demographics of the county [21]. There are conflicting viewpoints on the importance of race and ethnicity in breast cancer diagnosis and outcomes. Haque et al. proposed that the biological subtype of the cancer itself is more strongly correlated with the risk of subsequent breast cancer than a women’s race/ethnicity [22]. In contrast, other studies identified that ethnic minorities were more likely to report barriers to seeking help when compared with their white counterparts, creating a significant delay in diagnosis. This was due to the lack of awareness of signs and symptoms, lack of effective screenings, and different cultural beliefs about breast cancer [23,24]. African American women made up a disproportionately large percentage of patients diagnosed with breast cancer in the ED (22%), as compared with the percentage of African Americans in San Bernardino County (7%). Many studies have demonstrated the increased burden of breast cancer and subsequent mortality faced by African American women, due to both genetic predispositions and social inequality [25,26]. We noted that most of the patients diagnosed with breast cancer in the ED at ARMC were either Hispanic or African American, therefore many of our patients may also be affected by racial and social barriers to accessing healthcare [23,24,27].

It is important to explore possible solutions to minimize the health care disparities in the diagnosis and treatment of breast cancer. In the U.S., safety net providers exist as one solution to bridge the gap in care provided to individuals that are uninsured, vulnerable, or covered by Medicaid. [17] To help alleviate barriers to care among underserved women, several privately funded programs were developed such as the Avon Patient Navigation Program and the National Breast and Cervical Cancer Early Detection Program (NBCCEDP). These programs provide breast cancer screenings, treatments, transportation, access to appointments, financial help, bilingual interpreters, and care across the entire cancer continuum [28,29]. New healthcare legislation and regulations also impacted the screening and diagnosis of breast cancer among women. Adams et al. found that with the Breast and Cervical Cancer Prevention and Treatment Act of 2000, more women were screened for cancer and enrolled in Medicaid sooner, likely leading to earlier treatment of their cancer [30]. By increasing screening rates in this population and addressing the barriers to care with possible solutions, these patients may have an earlier diagnosis and a higher chance of survival [28,30].

This study has several limitations including the low number of patients and being a single center, retrospective study at a California county hospital. The lack of patients enrolled in this study limits its scope and power. The results reflect the demographics of San Bernardino County’s population, so they are not easily generalizable. Nevertheless, our findings explored the demographics of women who were first suspected to have breast cancer and had a final diagnosis by their PCP. This information may help design prospective studies to explore the different characteristics affecting the health care of women in our community.

Awareness of commonalities within this population of patients could help shape the formation of diagnostic algorithms that consider socioeconomic, cultural, and social demographics. These algorithms could then be used to help patients presenting to the ED with suspected breast cancer. These practices, along with extra screening and community education, could increase the chances of earlier detection of breast cancer in the ED and outpatient setting.

## Conclusions

This study reported that Hispanic and African American women had a higher likelihood of newly diagnosed breast cancer in the ED in our community. This may be due to the health care disparities in our community. Further research is needed to understand how these demographics can be used to detect breast cancer at an earlier stage, possibly by increasing access to primary care providers, regular screening, and education and prevention programs.
